# Retraction Note: Improved glycemic control, pancreas protective and hepatoprotective effect by traditional poly-herbal formulation “Qurs Tabasheer” in streptozotocin induced diabetic rats

**DOI:** 10.1186/s12906-022-03786-9

**Published:** 2022-11-17

**Authors:** Danish Ahmed, Manju Sharma, Alok Mukerjee, Pramod W. Ramteke, Vikas Kumar

**Affiliations:** 1 Department of Pharmaceutical Sciences, Faculty of Health Sciences, Sam Higginbottom Institute of Agriculture, Technology & Sciences (SHIATS)-Deemed University, Allahabad, Uttar Pradesh India; 2 Department of Pharmacology, Faculty of Pharmacy, Jamia Hamdard, New Delhi, India; 3 United Institute of Pharmacy, UCER, Allahabad, Naini India; 4 Department of Biological Sciences, Sam Higginbottom Institute of Agriculture, Technology & Sciences (SHIATS)-Deemed University, Allahabad, Uttar Pradesh India

**Retraction Note: BMC Complement Altern Med 13, 10 (2013)**

**https://doi.org/10.1186/1472-6882-13-10**

The Editor has retracted this article because there are concerns with the histological sections shown in Figure 10, specifically:

• Panels PN and PQ appear to show repeated areas between these two panels

• Panels PN, PQ50, PQ100 and PQ200 appear to show repeated areas within these panels

The authors have stated that they do not have access to the original data. The Editor therefore no longer has confidence in the results and conclusions reported. None of the authors has responded to the Editor about this retraction.

